# Human neural stem cells repress glioma cell progression in a paracrine manner by downregulating the Wnt/β‐catenin signalling pathway

**DOI:** 10.1002/2211-5463.13671

**Published:** 2023-07-26

**Authors:** Xiaolin Yin, Xiumei Liu, Xiangyi Xiao, Kaiyu Yi, Weigong Chen, Chao Han, Liang Wang, Ying Li, Jing Liu

**Affiliations:** ^1^ Stem Cell Clinical Research Center, National Joint Engineering Laboratory, Regenerative Medicine Center, The First Affiliated Hospital of Dalian Medical University Dalian Medical University China; ^2^ Dalian Innovation Institute of Stem Cell and Precision Medicine China

**Keywords:** conditioned medium, glioma, human neural stem cells, invasion, proliferation

## Abstract

Neural stem cells (NSCs) play crucial roles in neurological disorders and tissue injury repair through exerting paracrine effects. However, the effects of NSC‐derived factors on glioma progression remain unclear. This study aimed to evaluate the effects of human NSC‐conditioned medium (NSC‐CM) on the behaviour of glioma cells using an *in vitro* co‐culture system. Cell counting kit‐8 and 5‐ethynyl‐2′‐deoxyuridine assays revealed that NSC‐CM inhibited glioma cell proliferation and growth in a fetal bovine serum (FBS)‐independent manner. In addition, our wound‐healing assay demonstrated that NSC‐CM repressed glioma cell migration, while results from transwell and 3D spheroid invasion assays indicated that NSC‐CM also reduced the invasion capacity of glioma cells. Flow cytometry showed that NSC‐CM prevented cell cycle progression from the G1 to S phase and promoted apoptosis. Western blotting was used to show that the expression of Wnt/β‐catenin pathway‐related proteins, including β‐catenin, c‐Myc, cyclin D1, CD44 and Met, was remarkably decreased in NSC‐CM‐treated glioma cells. Furthermore, the addition of a Wnt/β‐catenin pathway activator, CHIR99021, significantly induced the expression of β‐catenin and Met and increased the proliferative and invasive capabilities of control medium‐treated glioma cells but not those of NSC‐CM‐treated glioma cells. The use of enzyme‐linked immunosorbent assays (ELISA) revealed the secretion of some antitumour factors in human and rat NSCs, including interferon‐α and dickkopf‐1. Our data suggest that NSC‐CM partially inhibits glioma cell progression by downregulating Wnt/β‐catenin signalling. This study may serve as a basis for developing future antiglioma therapies based on NSC derivatives.

## Graphical abstract

Human neural stem cell‐conditioned medium (hNSC‐CM) can inhibit glioma cell proliferation, invasion, migration and cell cycle progression *in vitro* while inducing apoptosis. Further experiments reveal that the addition of a Wnt/β‐catenin activator (CHIR99021) induces the expression of β‐catenin and Met and increases the proliferative and invasive capacities of control medium‐treated glioma cells but not those of hNSC‐CM‐treated glioma cells.

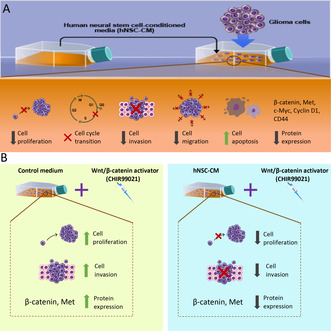

**Abbreviations**
bFGF: basic fibroblast growth factor
CCK‐8: cell counting kit‐8
DKK1: Dickkopf‐1
EdU: 5‐ethynyl‐2′‐deoxyuridine
EGF: epidermal growth factor
ELISA: enzyme‐linked immunosorbent assay
EMT: epithelial‐to‐mesenchymal‐like transition
FBS: fetal bovine serum
hMSCs: human mesenchymal stem cells
IFN‐α: interferon‐α
NPCs: neural progenitor cells
NSC‐CM: NSC‐conditioned medium
NSCs: neural stem cells
P/S: penicillin–streptomycin
PBS: phosphate‐buffered saline
PVDF: polyvinylidene fluoride membrane
RIPA: radioimmunoprecipitation assay
SVZ: subventricular zone

## Introduction

Neural stem cells (NSCs) are multipotent cells with strong self‐renewal capacities and the potential to differentiate into neurons, astrocytes and oligodendrocytes [1]. NSCs can replace injured cells, repair tissues and regulate neurological disorders such as neural injury, ischaemic stroke, brain inflammation and neurodegenerative diseases via their paracrine activities [2, 3, 4]. NSC‐derived factors possess anti‐inflammatory potential [5, 6, 7] and reduce inflammatory cytokine expression in activated macrophages and injured spinal cord tissues [8]. NSC‐conditioned medium (NSC‐CM) protects neurons and promotes propriospinal neurons to undergo neural circuit reconnection following spinal cord injury [9]. However, whether human NSC‐derived factors can regulate cancer progression remains unclear.

Gliomas are a group of cancers derived from alterations in NSCs, neural progenitor cells (NPCs), astrocytes and oligodendrocytes, generating astrocytomas (grade 2, 3, or 4), oligodendrogliomas (grade 2 or 3) and glioblastomas (grade 4) [10]. Glioblastoma is a highly malignant subtype with high rates of cell division and invasion and is associated with high recurrence and low 5‐year survival rates [11]. Some types of stem cells release secretome‐ and exosome‐mediated paracrine factors, which may greatly improve the effectiveness of current treatment strategies for glioblastoma [12]. For example, neural stem‐like cells generated from adult bone marrow can track migratory glioma cells and deliver IL‐23 *in situ*, which induces tumour‐specific antitumour activity [13]. Mesenchymal stem cell (MSC)‐secreted exosomes display antiglioma activity by regulating the PTENP1/miR‐10a‐5p/PTEN pathway [14]. Human induced NSCs possess tumour‐homing capacity and deliver therapeutic transgenes to inhibit the glioblastoma progression [15]. Therefore, NSCs can be used as vehicles to selectively deliver various anticancer agents to tumour sites [15, 16]. However, NSCs can also reportedly accelerate tumour formation, based on observations in the Ln229 glioblastoma cell line in nude mice [2]. MSCs from different sources can promote or suppress the growth of glioma cells under different conditions [17]. MSC‐conditioned medium impacts cell growth and the malignant progression of various types of cancers [18, 19, 20]. However, the effects of human NSC derivatives on glioma progression have not yet been elucidated.

The Wnt/β‐catenin pathway plays a critical role in malignant transformation and cancer progression [21]. β‐catenin is upregulated in various human cancers such as liver cancers [22] and gliomas [23]. Abnormal Wnt/β‐catenin signalling promotes cell proliferation and invasion and represses apoptosis during glioma development [24, 25, 26]. Inhibiting the effects of abnormal Wnt/β‐catenin signalling may be a vital step in preventing glioma progression. Rat embryonic neural stem/progenitor cell (NSC/NPC)‐conditioned medium inhibits U87 cell growth and invasion by downregulating β‐catenin expression [27]. However, the effects of human NSC‐CM on glioma cell progression remain unclear.

The aim of the present study was to investigate the effects of human NSC‐CM on different malignant glioma cell lines via an *in vitro* co‐culture system and to identify whether human NSC derivatives can regulate glioma cell progression through the Wnt/β‐catenin pathway. The results of the present study provide a foundation for the future development of human NSC‐derived factor‐based therapies for the targeted clinical treatment of gliomas.

## Materials and methods

### Cell culture

Hs683 cells (American Type Culture Collection, Manassas, VA, USA) and U87 cells (Shanghai HuicH Biotech Co., Ltd., Shanghai, China) were cultured in Dulbecco's Modified Eagle Medium (Gibco, Carlsbad, CA, USA) supplemented with 10% FBS (Gibco) and 1% penicillin–streptomycin (P/S) (Gibco). T98G cells (American Type Culture Collection) were cultured in Minimum Essential Medium (Gibco) supplemented with 10% FBS and 1% P/S. Human NSCs were isolated from the hippocampi of abortive foetuses and cultured *in vitro* in our laboratory in compliance with the Declaration of Helsinki and local ethical committees. The stemness features of the NSCs were identified by immunostaining them with antibodies against Sox2, Nestin, microtubule‐associated protein 2, glial fibrillary acidic protein and myelin basic protein as described previously [28]. Briefly, abortive foetuses were collected at 6–12 weeks of pregnancy. Fetal hippocampal tissue was separated, cut into small pieces and rinsed several times with precooled complete medium. NSCs isolated from the hippocampal tissues were cultured in Fetal Neural Stem Cell Complete Culture Medium (Cyagen Biosciences Inc., Guangzhou, China). In our previous study, normal rat embryonic NSCs were obtained and cultured, and their viability, self‐renewal, proliferation and differentiation capacities were determined [29]. The rat NSCs were cultured in Dulbecco's Modified Eagle Medium/Nutrient Mixture F12 (Gibco) supplemented with mitogenic factors [20 ng·mL^−1^ EGF (Gibco) and bFGF (Gibco)] to form neurospheres. To investigate whether FBS supplementation affects the inhibitory effect of NSC‐CM on glioma cell growth, we cultured glioma cells in NSC‐CM and control medium supplemented with different ratios (10%, 5% and 1%) of FBS. All cell lines were cultured at 37 °C in the presence of 5% CO_2_. CHIR99021 reagent (MedChemExpress, Princeton, NJ, USA) was added to the NSC‐CM or control medium (used to treat the glioma cells for 3 days at the concentration of 10 μm) to activate the Wnt/β‐catenin pathway.

### Collection of NSC‐conditioned medium (NSC‐CM)

Human and rat NSC‐CM was harvested from the NSC culture system on the seventh day, filtered using a 0.22 μm syringe filter and then stored at −80 °C until use. Fresh NSC culture medium was used as a control. Glioma cells were cultured in either NSC‐CM or control NSC medium for subsequent biological behavioural assays.

### Cell counting kit‐8 (CCK‐8) assay

The effects of NSC‐CM on glioma cell proliferation were evaluated using CCK‐8 reagent (Sigma‐Aldrich, St. Louis, MO, USA) in accordance with the manufacturer's instructions. Briefly, 2 × 10^3^ cells per well were seeded in 96‐well plates, incubated for 6 h, washed with phosphate‐buffered saline (PBS) and then cultured in either NSC‐CM or control NSC medium. CCK‐8 solution was added to each well, and the wells were co‐cultured at 37 °C for 2 h to measure cell proliferation. Absorbance was detected at 450 nm with a Universal Microplate Reader (Bio Tek, Winooski, VT, USA).

### 5‐ethynyl‐2′‐deoxyuridine (EdU) assay

Cell proliferation was observed using a Click‐iT EdU Labelling kit (Thermo Fisher Scientific, Shanghai, China) in accordance with the manufacturer's protocol. Briefly, 2 × 10^4^ cells were seeded in a confocal dish. Six hours later, NSC‐CM or control NSC medium was added, and the cells were cultured for 3 days before being incubated with 10 μm EdU for 2 h at 37 °C. Nuclear staining was performed using 250 μL of 1 × HCS Nuclear Mask Blue stain solution for 0.5 h at room temperature. The cells were scanned using a CQ1 Confocal Quantitative Image Cytometer (Yokogawa, Kanazawa, Japan). The proliferation index was determined by quantifying the percentage of EdU‐labelled cells using CQ1 Software (Yokogawa, Kanazawa, Japan).

### Wound‐healing assay

The migration capabilities of the cells were assessed using a wound healing assay. Approximately 1 × 10^6^ cells per well were seeded in 6‐well plates and serum‐starved overnight. The next day, the cells were scratched with a sterile 200 μL pipette tip, washed with PBS and then incubated in either NSC‐CM or control NSC medium. After scratching, the gap closure process was photographed at 0, 12 and 24 h. Wound closure was measured using imagej (https://imagej.nih.gov/ij/download.html), and the proportion of wound closure was assessed as: ((original gap distance − gap distance at 12 or 24 h)/original gap distance) × 100%.

### Cell apoptosis analysis

Cell apoptosis assays were performed using a BD FACS Calibre flow cytometer (BD, New York, NY, USA). Briefly, 1 × 10^5^ cells were seeded in a 10‐cm dish containing a regular complete medium. After 6 h, cells were treated with either NSC‐CM or control NSC culture medium for various durations. The cells were then harvested, washed in PBS and stained using an APC Annexin V Apoptosis Detection Kit with PI (BioLegend, San Diego, CA, USA) for 10 min. Early and late apoptotic cells were quantified using a flow cytometer.

### Cell cycle analysis

Flow cytometry was conducted to investigate whether NSC‐CM inhibits glioma cell proliferation via cell cycle arrest. After being co‐cultured in NSC‐CM and control medium for 3 days, glioma cells were resuspended twice in precooled PBS. Next, 300 μL of the resuspended cells was fixed by adding 700 μL of precooled ethanol dropwise. The cells were centrifuged at 250 ***g*** for 5 min, washed twice in PBS and stained with FxCycle PI/RNase Staining Solution (Thermo Fisher Scientific) at room temperature in the dark for 30 min. The DNA content was analysed by flow cytometry using FCS Express Research Edition software (BD).

### Transwell invasion assay

Transwell and three‐dimensional (3D) spheroid invasion assays were performed to investigate whether human NSC‐CM impacts glioma cell invasion. Cell invasion assays were performed using 24‐well Transwell Permeable Supports (Corning Inc., Corning, NY, USA). The inserts were coated with diluted Matrigel (BD) in accordance with the manufacturer's instructions. Cells were treated with either NSC‐CM or control NSC medium for 3 days before being transferred to the upper Matrigel chamber, while the lower chamber was filled with culture medium containing 20% FBS. The cells were incubated at 37 °C for 30 h in the presence of 5% CO_2_. Invasive glioma cells were fixed with 4% paraformaldehyde solution, stained with 0.1% crystal violet, photographed and enumerated [30].

### 3D spheroid invasion assay

The 3D spheroid invasion model is an excellent *in vitro* system to mimic the *in vivo* environment of a human body [31]. We evaluated the effects of NSC‐CM on the invasive capacity of glioma cells. To generate a 3D spheroid model, 5 × 10^3^ glioma cells were added to the lid of a 10‐cm dish and cultured for 3 days until cell spheroids formed. Each cell spheroid was coated with 20 μL of 1.5 mg mL^−1^ Rat Tail Tendon Collagen Type I (Solarbio, Beijing, China). The collagen‐coated spheroids were removed, placed in a 12‐well plate and then cultured in either NSC‐CM or control NSC medium for 24 h. Invasive cells around the spheroid were photographed under a microscope. The distances travelled by the invasive cells (from the centre of the 3D spheroid) were quantified using imagej.

### Western blotting assay

Western blotting was performed as described previously [32] to investigate whether human NSC‐CM exerts its' antitumour effects by regulating the Wnt/β‐catenin signalling pathway. Total proteins were lysed in cold radioimmunoprecipitation assay (RIPA) lysis buffer containing protease and phosphatase inhibitors (Sigma). Briefly, cells were harvested, rinsed with cold PBS and lysed with RIPA solution for 30 min. The cell lysates were centrifuged at 18,700 *g* for 15 min at 4 °C and then quantified using a bicinchoninic acid assay. The protein samples were then separated by NuPAGE Novex 4‐12% Bis‐Tris gel (Invitrogen, Carlsbad, CA, USA) electrophoresis and transferred onto polyvinylidene fluoride (PVDF) membranes (Merck Millipore, Billerica, MA, USA). The membranes were incubated at 4 °C overnight with antibodies against GAPDH (1 : 8000, Cell Signalling Technology, Danvers, MA, USA), Met (1 : 1000, Cell Signalling Technology), CD44 (1 : 1000, Cell Signalling Technology), c‐Myc (1 : 1000, Cell Signalling Technology), β‐catenin (1 : 1000, Cell Signalling Technology) and cyclin D1 (1 : 1000, Cell Signaling Technology). The next day, the PVDF membranes were incubated with a horseradish peroxidase‐labelled secondary antibody for 1 h at room temperature and then detected using a chemiluminescence analysis system.

### Enzyme‐linked immunosorbent assay (ELISA)

To investigate whether antitumour factors are present in human and rat NSC‐CM, we screened secretions as previously described [33, 34] and detected them using ELISA. Specifically, interferon‐α (IFN‐α) and dickkopf‐1 (DKK1) concentrations were detected in human and rat NSC‐CM using an ELISA kit (Spbio, Wuhan, China) according to the manufacturer's instructions. The optical density values were measured at 450 nm.

### Statistical analysis

All analyses were performed with data from three independent biological repeats, and data are presented as the mean ± standard deviation (SD). Comparisons between two groups were performed using Student's *t*‐tests (unpaired, two‐tailed). Statistical analyses were conducted using graphpad prism 6 for Windows (GraphPad, San Diego, CA, USA) and imagej. Statistical significance was set as follows: ns, not significant; *P < 0.05; **P < 0.001.

### Ethical approval statement

All patients signed an informed consent form and agreed to be donors. This study was approved by the Ethics Committee of the First Affiliated Hospital of Dalian Medical University (Ethics Reference No: PJ‐KS‐KY‐2019‐31(X)) and followed the guidelines set by the Declaration of Helsinki.

## Results

### NSC‐CM inhibits glioma cell proliferation in an FBS‐independent manner

**Fig. 1:**
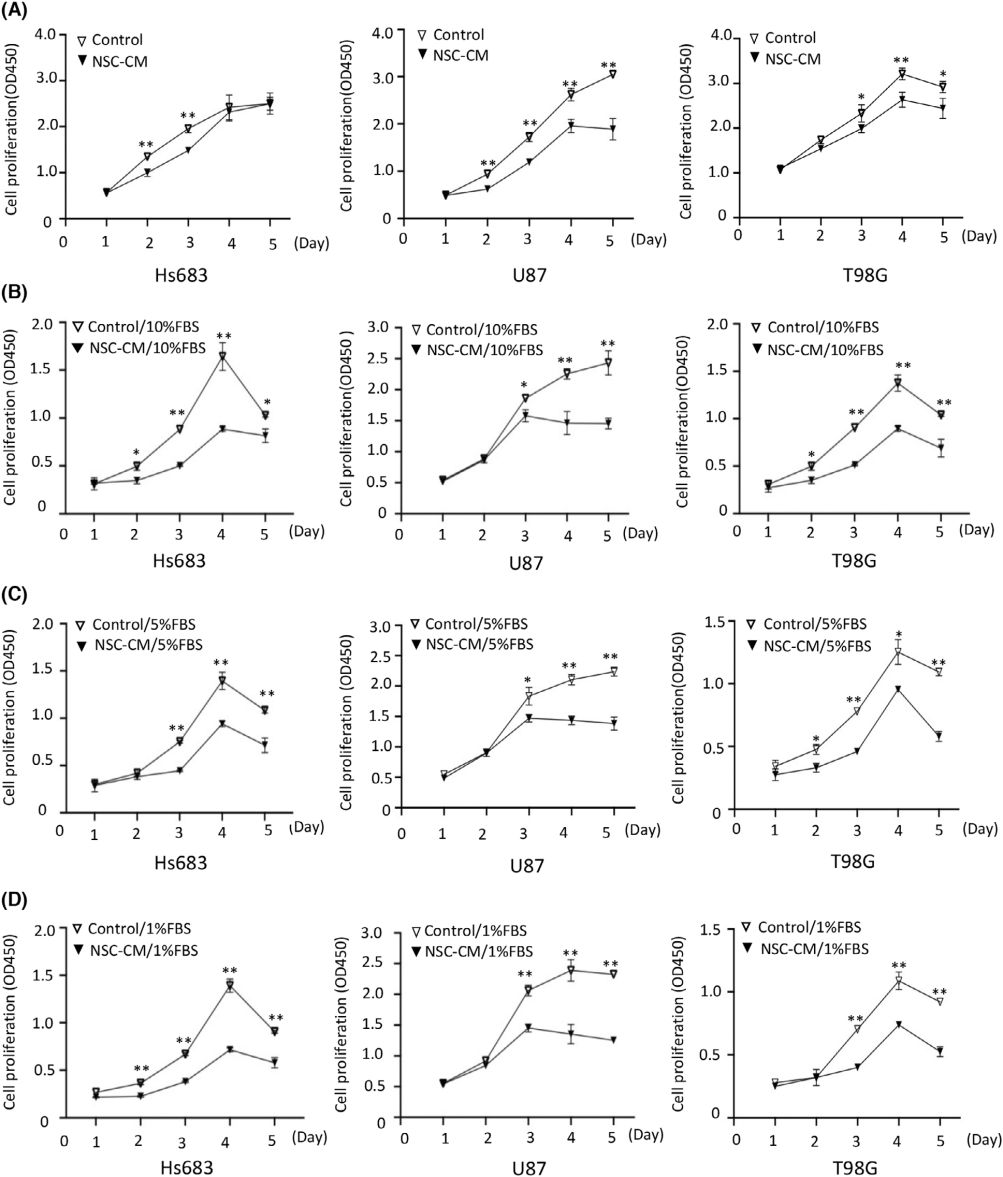
NSC‐CM inhibits glioma cell proliferation in an FBS‐independent manner. Growth curves of Hs683, U87 and T98G cell lines treated with (A) NSC‐CM and control medium, (B) NSC‐CM and control medium supplemented with 10% FBS, (C) NSC‐CM and control medium supplemented with 5% FBS, or (D) NSC‐CM and control medium supplemented with 1% FBS. NSC‐CM refers to neural stem cell‐conditioned medium. Control medium refers to fresh medium for the NSCs. All data are presented as the means ± SD; *n* = 3; Student's *t*‐tests. *P < 0.05; **P < 0.001. FBS, fetal bovine serum; NSC‐CM, neural stem cell‐conditioned medium; SD, standard deviation.

**Fig. 2:**
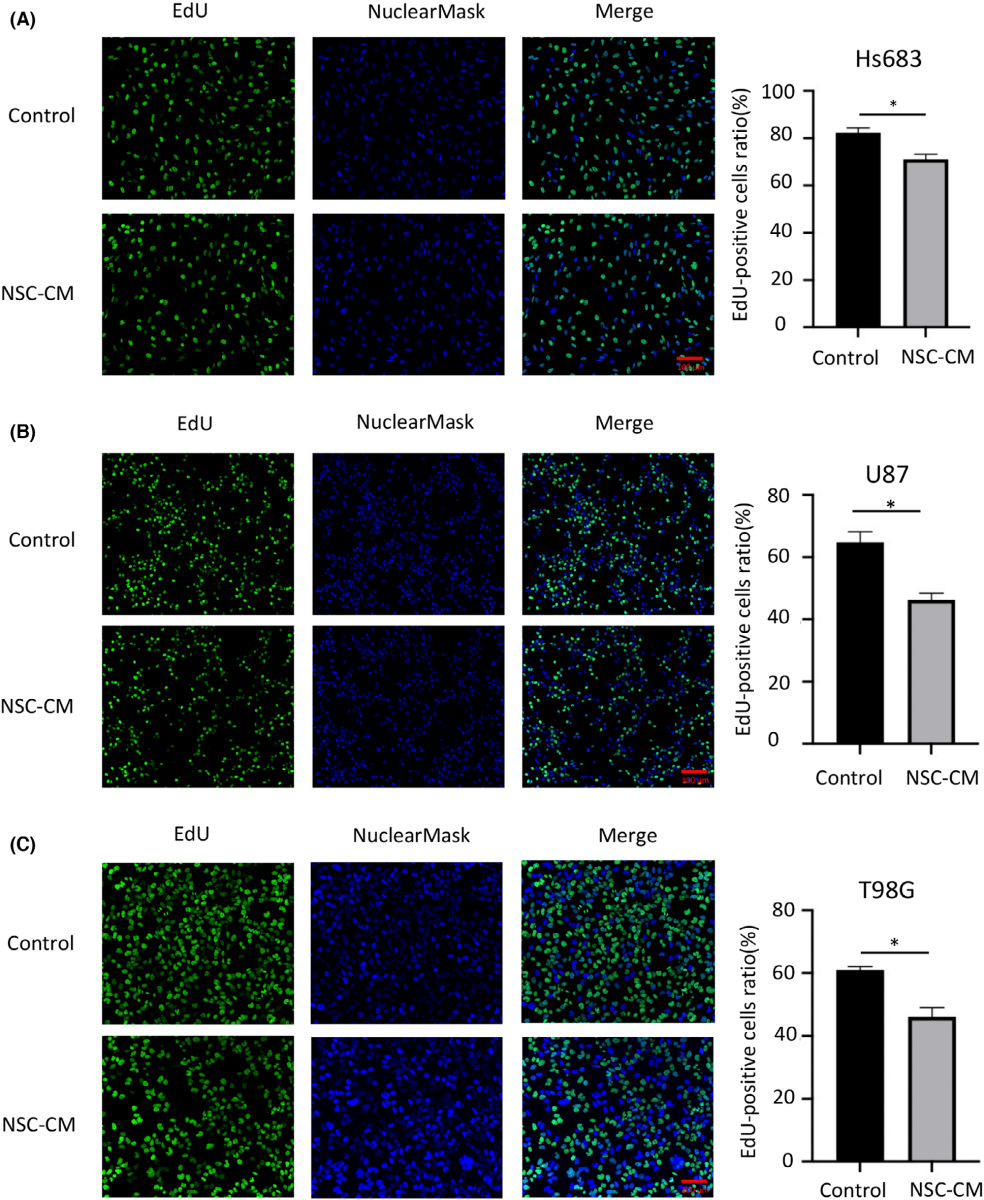
NSC‐CM reduces glioma cell proliferation based on an EdU assay. Proliferation of (A) Hs683, (B) U87 and (C) T98G cells treated with NSC‐CM or control medium (left). Rates of EdU‐positive glioma cells were quantified using imagej (right). NSC‐CM refers to neural stem cell‐conditioned medium. Control medium refers to the fresh medium for NSCs. Scale bar: 100 μm. All data are presented as the means ± SD; *n* = 3; Student's *t*‐tests. *P < 0.05. EdU, 5‐ethynyl‐2′‐deoxyuridine; NSC‐CM, neural stem cell‐conditioned medium; SD, standard deviation.

The CCK‐8 and EdU assay results showed that the glioma cell growth curves accelerated with time, peaked on the 3rd or 4th day and then declined on the 5th day. A significant difference in growth was observed between glioma cells treated with NSC‐CM and control medium after 3 days (Fig. 1A). Moreover, the rates of EdU‐positive cells in Hs683, U87 and T98G cells treated with NSC‐CM were 11%, 18% and 15% lower than those in cells treated with the control medium, respectively (Fig. 2A–C). These results indicate that NSC‐CM can repress glioma cell proliferation.

Meanwhile, the proliferation of the glioma cells treated with NSC‐CM supplemented with 10%, 5% and 1% FBS was significantly lower than that of cells treated with the control medium (Fig. 1B–D). These results indicate that NSC‐CM inhibits glioma cell proliferation in an FBS‐independent manner.

### NSC‐CM represses glioma cell migration and invasion

**Fig. 3:**
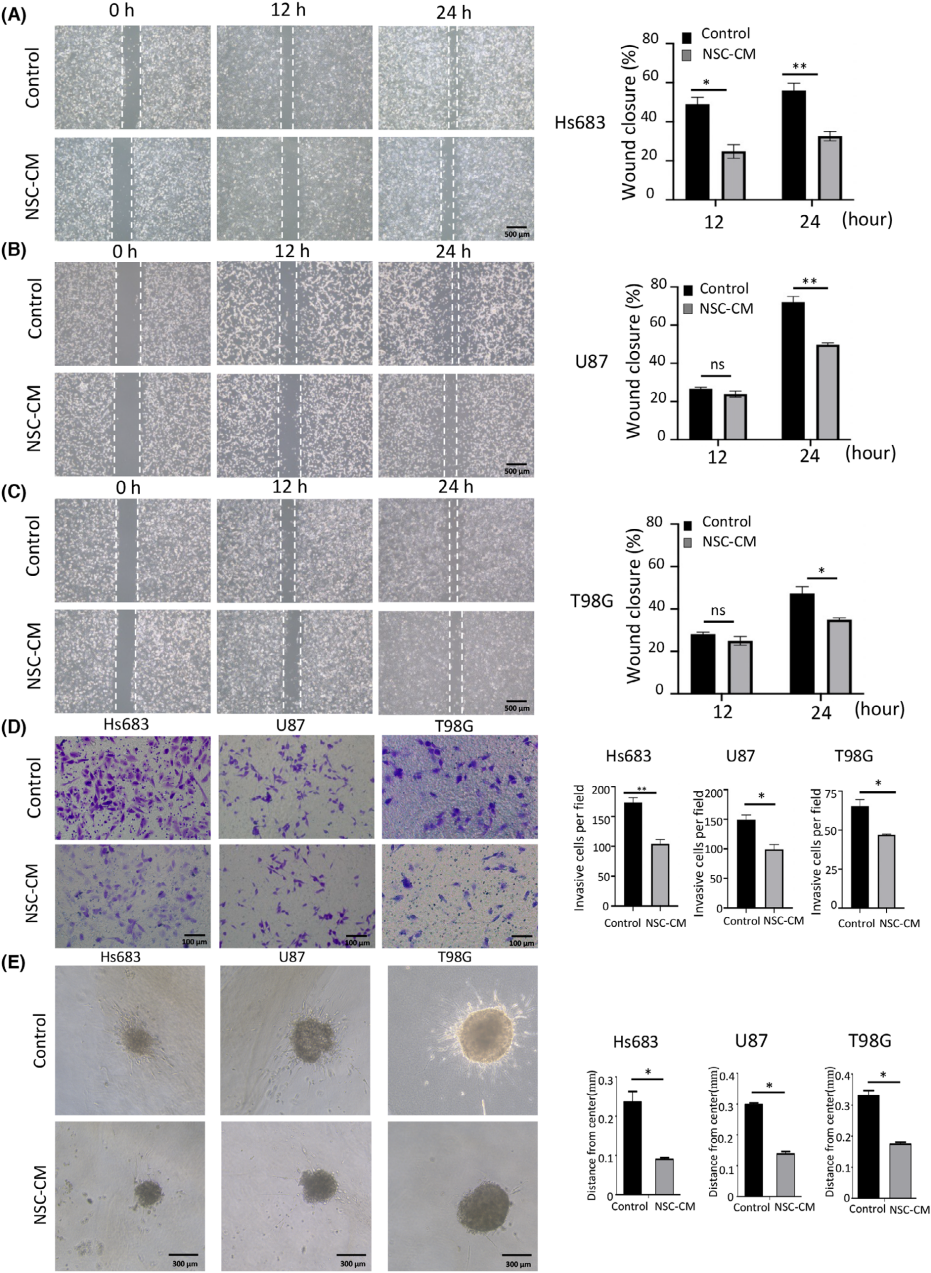
NSC‐CM represses glioma cell migration and invasion. Wound closure in (A) Hs683, (B) U87 and (C) T98G cells treated with NSC‐CM or control medium. (D) Transwell invasion in Hs683, U87 and T98G cells treated with NSC‐CM or control medium (left). The number of invasive cells per field was quantified in both NSC‐CM and control medium (right). (E) Three‐dimensional spheroid invasion in Hs683, U87 and T98G cells treated with NSC‐CM or control medium (left). The distances travelled by the invasive Hs683, U87 and T98G cells (from the centre of the 3D spheroid) were quantified using imagej (right). NSC‐CM refers to neural stem cell‐conditioned medium. Control medium refers to the fresh medium for NSCs. Scale bar: 500 μm (A–C); 100 μm (D); 300 μm (E). All data are presented as the means ± SD; *n* = 3; Student's *t*‐tests. *P < 0.05; **P < 0.001; ns, not significant. NSC‐CM, neural stem cell‐conditioned medium; SD, standard deviation.

Wound‐healing assay results showed that wound closure was 23%, 22% and 12% lower in Hs683, U87 and T98G cells treated with NSC‐CM, respectively, than in those treated with the control medium (Fig. 3A–C). These results indicate that NSC‐CM can repress glioma cell migration *in vitro*.

Transwell invasion assay results showed that the invasive capacity of glioma cells treated with NSC‐CM was significantly lower than that of cells treated with the control medium (Fig. 3D). In addition, 3D spheroid invasion assay results showed that the invasion distance (from the 3D spheroid centre) was significantly shorter in glioma cells treated with NSC‐CM than in those treated with the control medium (Fig. 3E). These data suggest that NSC‐CM represses glioma cell invasion *in vitro*.

### NSC‐CM inhibits the glioma cell cycle and induces cell apoptosis

**Fig. 4:**
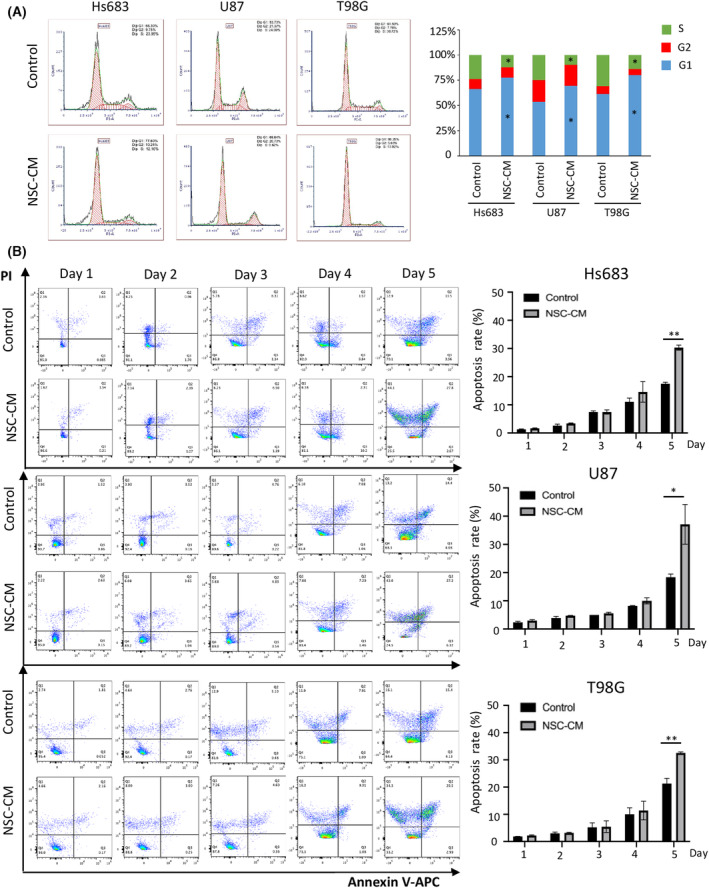
NSC‐CM restrains the glioma cell cycle and induces cell apoptosis. (A) Cell cycle of Hs683, U87 and T98G cells treated with NSC‐CM or control medium (left). The ratios of Hs683, U87, and T98G cells in the S, G1 and G2 phases were quantified after treatment with either NSC‐CM or control medium (right). (B) Apoptosis assay results for Hs683, U87 and T98G cells treated with NSC‐CM or control medium (left). The apoptosis rates of Hs683, U87 and T98G cells treated with NSC‐CM or control medium were quantified (right). NSC‐CM refers to neural stem cell‐conditioned medium. Control medium refers to the fresh medium for NSCs. All data are presented as the means ± SD; *n* = 3; Student's *t*‐tests. *P < 0.05; **P < 0.001. NSC‐CM, neural stem cell‐conditioned medium; SD, standard deviation.

The results of flow cytometry showed that NSC‐CM treatment for 3 days significantly increased the ratio of glioma cells in the G1 phase, while significantly decreasing the ratio of those in the S phase (Fig. 4A). These results suggest that NSC‐CM inhibited the G1‐to‐S transition.

Moreover, NSC‐CM treatment increased the number of early and late apoptotic glioma cells in a time‐dependent manner. No significant differences in apoptosis rate were observed between the glioma cells treated with NSC‐CM and control medium in the first 4 days (Fig. 4B). After 5 days, the apoptosis rates of the Hs683, U87 and T98G cells treated with NSC‐CM were 30.47%, 33.52% and 32.49%, compared with 17.06%, 18.48% and 19.53% in cultures grown in control medium, respectively (Fig. 4B). Interestingly, necrosis was also increased on the 5th day (12.9% of Hs683 cells grown in the control medium vs 44.1% in NSC‐CM; 13.2% of U87 cells grown in control medium vs 42.0% in NSC‐CM; 16.1% of T98G cells grown in the control medium vs 34.3% in NSC‐CM) (Fig. 4B). These results suggest that NSC‐CM can induce glioma cell apoptosis *in vitro*.

### NSC‐CM inhibits Wnt/β‐catenin signalling in glioma cells

**Fig. 5:**
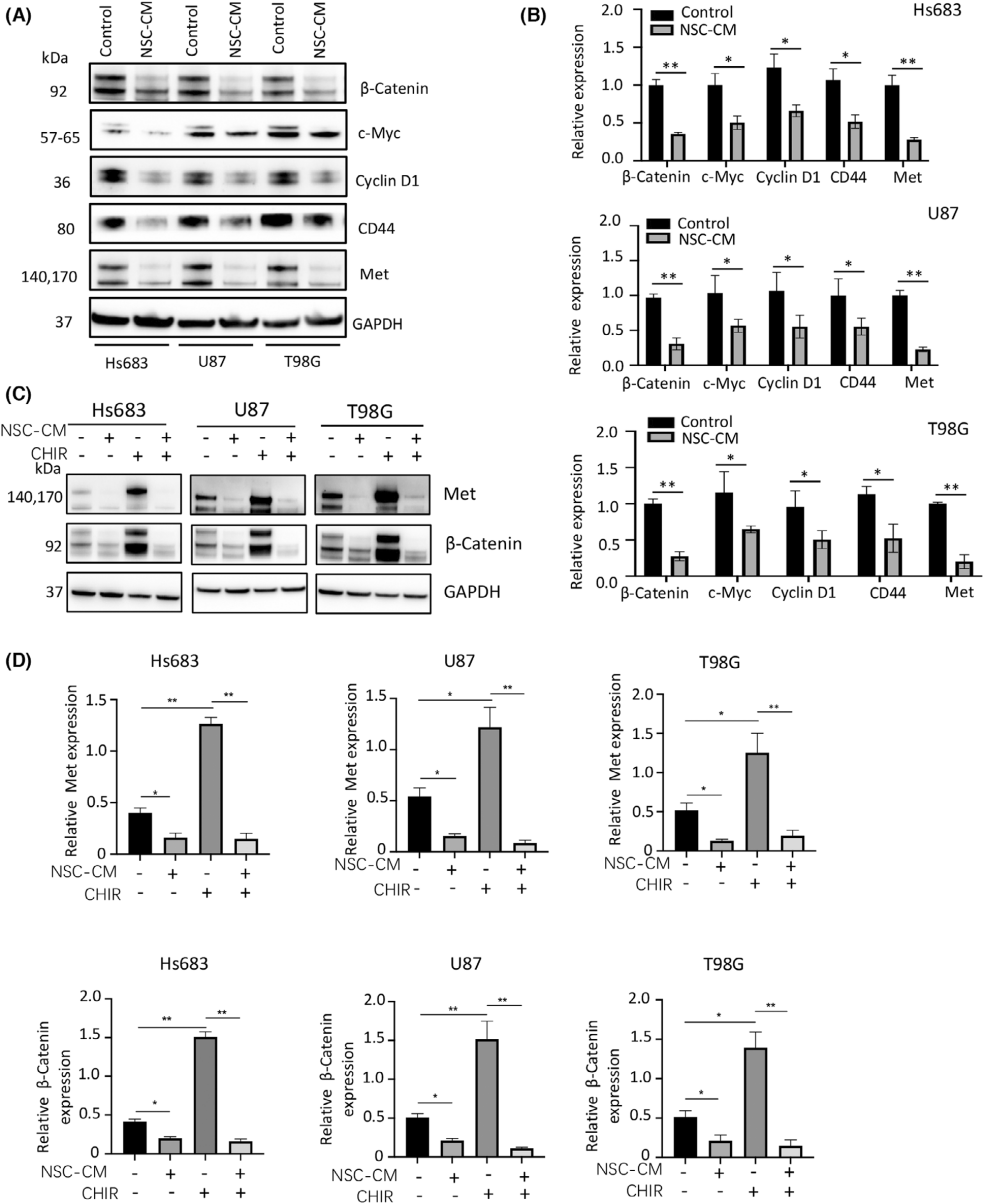
NSC‐CM represses Wnt/β‐catenin signalling in glioma cells. (A) Protein expression in Hs683, U87 and T98G cells treated with NSC‐CM or control medium based on western blotting results. (B) Relative protein expressions in Hs683, U87 and T98G cells showed in (A) were quantified using imagej. (C) β‐catenin and Met expression in Hs683, U87 and T98G cells treated with NSC‐CM or control medium supplemented with 10 μm CHIR99021 (activator of Wnt/β‐catenin pathway) for 3 days. (D) Relative expression of β‐catenin and Met in Hs683, U87 and T98G cells showed in (C) were quantified using imagej. NSC‐CM refers to neural stem cell‐conditioned medium. Control medium refers to the fresh medium for NSCs. All data are presented as the means ± SD; *n* = 3; Student's *t*‐tests. *P < 0.05; **P < 0.001. NSC‐CM, neural stem cell‐conditioned medium; SD, standard deviation.

**Fig. 6:**
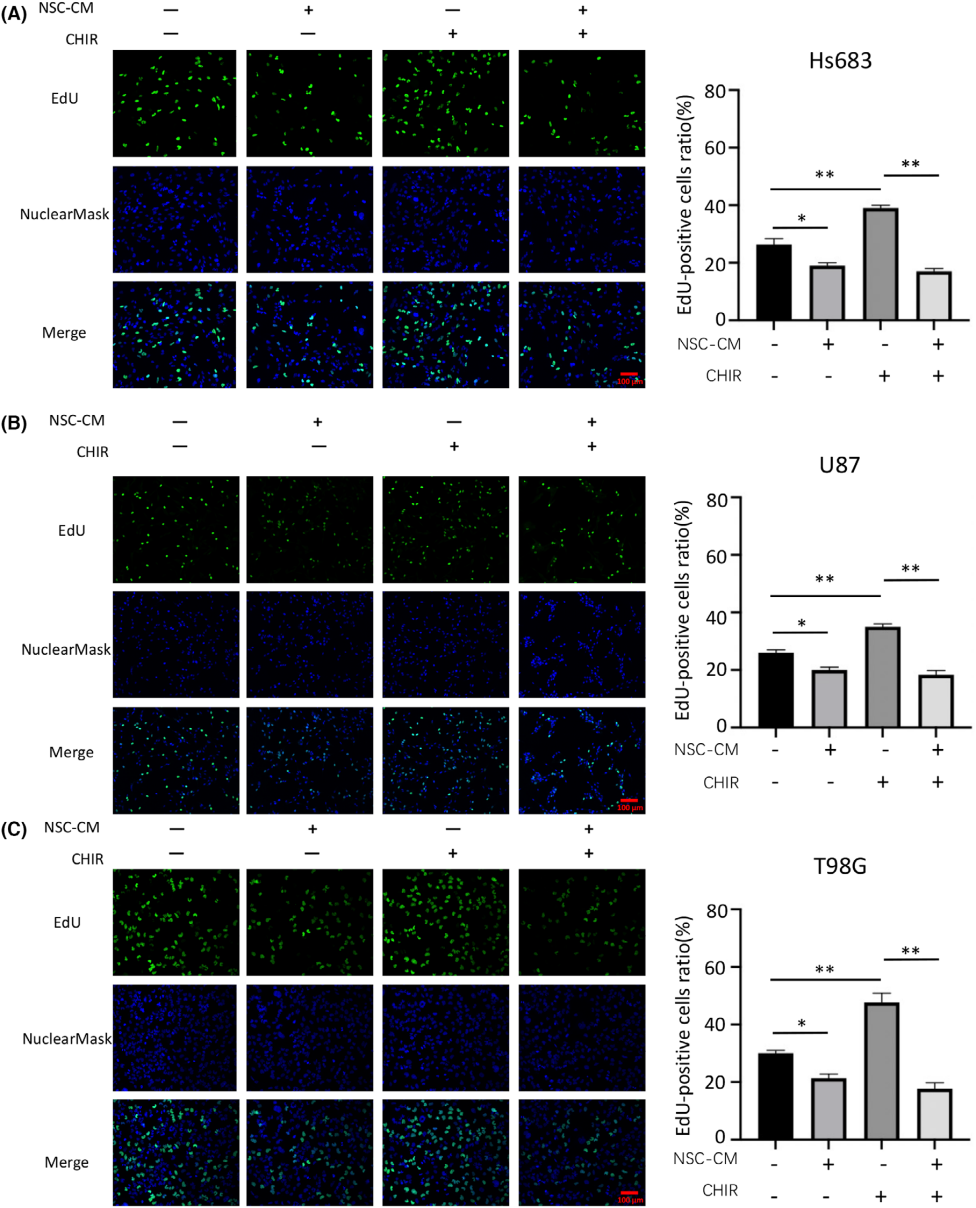
NSC‐CM represses glioma cell proliferation by inactivating Wnt/β‐catenin signalling. Proliferation of (A) Hs683, (B) U87 and (C) T98G cells treated with control medium, NSC‐CM or media supplemented with 10 μm CHIR99021 (Wnt/β‐catenin pathway activator) for 3 days (left). Rates of EdU‐positive glioma cells were quantified using imagej (right). NSC‐CM refers to neural stem cell‐conditioned medium. Scale bar: 100 μm. Control medium refers to the fresh medium for NSCs. All data are presented as the means ± SD; *n* = 3; Student's *t*‐tests. *P < 0.05; **P < 0.001. EdU, 5‐ethynyl‐2′‐deoxyuridine; NSC‐CM, neural stem cell‐conditioned medium; SD, standard deviation.

**Fig. 7:**
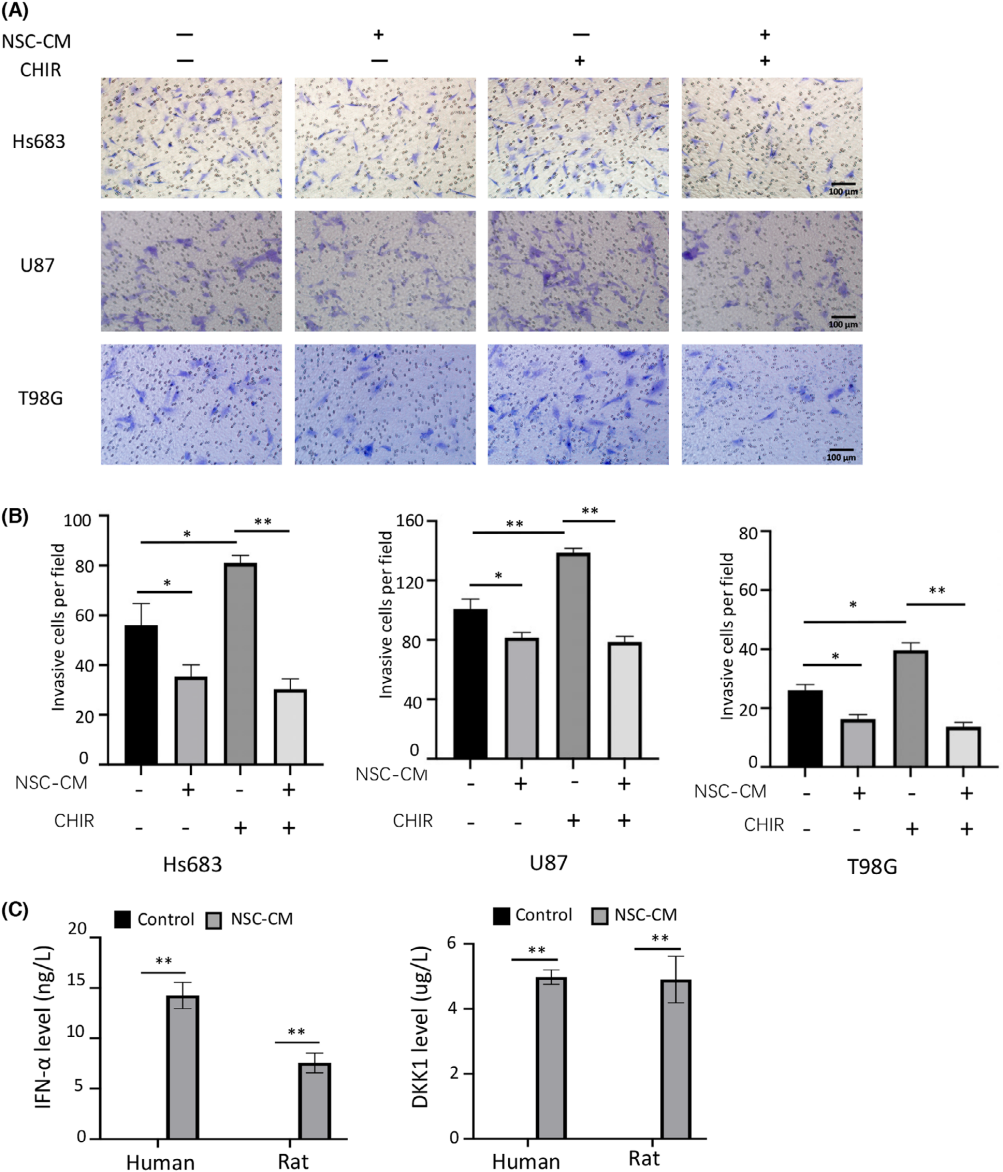
NSC‐CM represses glioma cell invasion by inactivating the Wnt/β‐catenin signalling pathway. (A) Invasion of Hs683, U87 and T98G cells treated with control medium, NSC‐CM or media supplemented with 10 μm CHIR99021 for 3 days (up). (B) Number of invasive Hs683, U87 and T98G cells per field was quantified (down). (C) ELISA assay of IFN‐α and DKK1 expression in human and rat NSC‐CM. NSC‐CM refers to neural stem cell‐conditioned medium from human or rat NSCs. Control medium refers to the fresh medium for NSCs. Scale bar: 100 μm. All data are presented as the means ± SD; *n* = 3; Student's *t*‐tests. *P < 0.05; **P < 0.001. DKK1, dickkopf‐1; ELISA, enzyme‐linked immunosorbent assay; IFN‐α, interferon‐α; NSC‐CM, neural stem cell‐conditioned medium; SD, standard deviation.

The results of western blotting showed that the expression of Wnt/β‐catenin signalling pathway‐related proteins, including β‐catenin, c‐Myc, cyclin D1, CD44 and Met, was significantly lower in glioma cells treated with NSC‐CM than in those treated with the control medium (Fig. 5A,B). Moreover, the addition of CHIR99021 (10 μm, for 3 days) significantly induced β‐catenin and Met expression in control medium‐treated glioma cells, but not in NSC‐CM‐treated glioma cells (Fig. 5C,D). However, it did not affect the expression of c‐Myc, cyclin D1 and CD44 (data not shown). Furthermore, the addition of CHIR99021 (10 μm, for 3 days) significantly increased the proliferation and invasion capabilities of control medium‐treated glioma cells but not those of NSC‐CM‐treated glioma cells (Figs 6A–C and 7A,B). Collectively, our data indicate that NSC‐CM inhibits glioma progression at least partially by inactivating the Wnt/β‐catenin signalling pathway.

### Comparison of antitumour factors from human and rat NSC‐CM by ELISA

The ELISA results showed that IFN‐α and DKK1 were present in both human and rat NSC‐CM (Fig. 7C). These results indicate that NSCs may release antiglioma factors into NSC‐CM to repress glioma cell progression.

## Discussion

The tumour microenvironment is complex and includes various immune cells, cytokines and growth factors [35]. The crosstalk between human NSC and glioma cells has not yet been fully elucidated. In the present study, we found that human NSC‐CM can repress the proliferation, migration and invasion capabilities of various glioma cell lines, while also inhibiting cell cycle progression and inducing glioma cell apoptosis. The antitumour effect of human NSC‐CM could be partially attributed to inactivation of the Wnt/β‐catenin signalling pathway. Furthermore, antitumour factors such as IFN‐α and DKK1 were observed in both human and rat NSC‐CM. Our findings provide a basis for future methods to inhibit glioma cell progression and will potentially advance the development of NSC‐derived antiglioma therapies.

Neural stem cells play a critical role in the tumour microenvironment via their paracrine activities [36, 37]. NSC‐derived exosomes inhibited mouse glioma cell growth by transporting oligonucleotides to a FLOT2 target [38]. Exosomal lncRNA PTENP1 mediates cell communication and exerts antitumour activity [14]. Numerous studies have shown that rat NSC‐CM‐induced antiglioma effects are related to the mitogen‐activated protein kinase (MAPK) [39, 40] and PI3K/AKT signalling pathways [27]. The present study demonstrated that human NSC‐CM represses glioma cell progression by downregulating the Wnt/β‐catenin pathway, which is consistent with previous reports. However, a previous study showed that NSCs can also accelerate tumour formation in nude mice by co‐culturing NSCs and Ln229 cells in a neurobasal medium [2]. This discrepancy may be attributed to the different glioma cell lines, NSC origins and experimental designs utilized between studies. The present study isolated human NSCs from abortive fetal hippocampi at 6–12 weeks of pregnancy. By contrast, the NSCs used in previous studies [2] were obtained from the subventricular zone (SVZ). A recent study has shown that NSCs in human SVZ tissue are the cells of origin of glioblastomas [41] and may contribute to relapse. In addition, the glioma cells were treated with NSC‐CM in our study, whereas they were simultaneously cultured with glioblastoma cells (in a 1 : 4 ratio) in the previous study [2].

Notably, adding CHIR99021 (10 μm, for 3 days) to the control medium significantly induced β‐catenin and Met expression in glioma cells (Fig. 5C,D) but did not affect the expression of c‐Myc, cyclin D1 and CD44 (data not shown). These results indicate that human NSC‐CM regulates the expression of c‐Myc, cyclin D1 and CD44 via a separate mechanism. β‐catenin activation has been implicated in the transformation, proliferation and invasion of various cancers [21]. The Wnt/β‐catenin pathway induces Met overexpression in colorectal cancer [42]. Met can drive cell migration and growth during embryogenesis and contributes to cell proliferation, invasion and metastasis in many cancers [43]. In the present study, human NSC‐CM inhibited glioma cell proliferation and invasion, at least partially by downregulating β‐catenin and Met. Cyclin D1 acts as a critical regulator of the G1/S cell cycle transition [44]. NSC‐CM‐mediated inhibition of cyclin D1 may cause cell cycle arrest and inhibit cell proliferation. CD44 overexpression could be relevant in regulating the highly invasive behaviour of gliomas [45], and NSC‐CM‐mediated CD44 inhibition may reduce the invasive capabilities of glioma cell lines. Furthermore, c‐Myc plays critical roles in cell proliferation, differentiation, cell cycle progression, metabolism and apoptosis [46]. According to a previous review [47], c‐Myc dysregulation can either promote proliferation or induce apoptosis depending on the *in vivo* cellular context. There are usually two main conditions under which c‐Myc would induce apoptosis. First, c‐Myc can induce apoptosis when the extracellular microenvironment lacks growth factors and other nutrients. Based on this condition, we speculated that by the 5th day, both the control medium and NSC‐CM became equally deficient in nutrients such as growth factors, and therefore, c‐Myc was able to induce apoptosis of glioma cells. Second, c‐Myc can induce apoptosis when pro‐apoptotic molecules become dominant over antiapoptotic molecules within the cell. In accordance with this notion, we speculated that on the 5th day, the pro‐apoptotic molecules secreted by NSCs in the NSC‐CM further promoted the apoptosis of glioma cells. This resulted in the higher apoptosis rate in glioma cells cultured in NSC‐CM than that in cells cultured in the control medium. In addition, on the 5th day, the cell necrosis rate of both the experimental and control groups was significantly increased compared with that observed on the first 3 days. This result is consistent with the decline seen in the growth curves on day 5 (Fig. 1A).

The capacity for migration is a malignant characteristic of cancer cells and correlates to higher mortality rates. In this study, glioma cells cultured in human NSC‐CM exhibited markedly less migration than those cultured in the control medium (Fig. 3A–C), and this result was consistent with that of a previous report [27]. However, some studies have shown that MSCs promote EMT in breast cancer [48]. The molecules secreted by rat adipose‐derived stem cells induce EMT‐like transformation in C6 cancer cells [13]. The inconsistency in the effects of various stem cells on glioma cell migration could be attributed to the complexity/heterogeneity of stem cell‐secreted paracrine factors, which may differentially modulate the behaviour of glioma cells [17, 49]. In addition, the study design, stem cell origins, culture conditions and crosstalk between cancer and stem cells may affect their antitumour properties.

To identify whether common antitumour factors exist in conditioned media derived from different NSCs, we detected secretions with potential antitumour activity [33, 34]. Notably, IFN‐α and DKK1 were detected in human and rat NSC‐CM (Fig. 7C). DKK1 antagonizes the Wnt signalling pathway by competing for binding to the Wnt‐assisted receptor LRP6 [50]. Human umbilical cord‐derived MSCs inhibit C6 glioma cell growth by secreting DKK1 [51]. Baicalin reduces the expression of β‐catenin and c‐Myc by promoting DKK1 expression [52]. Therefore, the inactivation of Wnt/β‐catenin signalling by NSC‐CM could be attributed to NSC‐released DKK1 in the tumour microenvironment. Type I IFNs can induce tumour cell apoptosis, repress tumour cell proliferation and metastasis, and activate antitumour immune responses [53, 54]. In the present study, IFN‐α was found to be expressed in human and rat NSC‐CM, suggesting that IFN‐α exerts antitumour activity by regulating immune cells in the tumour microenvironment. Collectively, the antiglioma effects of NSCs could be mediated by multiple paracrine factors through various mechanisms, which can provide alternative strategies for treating glioma. Although increasing evidence demonstrates that NSCs and their derived factors exhibit antitumour activities, the contributions of specific paracrine factors from different NSC origins remain largely unclear.

Studies on genetically engineered mouse models have shown that gliomas most likely originate from NSCs/NPCs in the SVZ and oligodendrocyte precursor cells [41, 55, 56]. A recent study using direct molecular genetic evidence from both patients and mouse models confirmed that NSCs in SVZ tissues are the cells of origin that harbour driver mutations in glioblastoma [41]. Therefore, normal NSCs from patients with glioblastoma are unsuitable for use in clinical treatment. Normal NSCs and their derivatives are expected to serve antitumour functions because of their low immunogenicity. Notably, the stemness features, proliferation, differentiation and specificity of NSCs change in an age‐dependent manner. A review demonstrated an age‐related decrease in the release of nucleotides from astrocytes [57]. Another review showed that NSCs and their progenitors exhibit reduced proliferation and neuronal production with age [58]. A key limitation of the present study is that the direct molecular mechanisms via which NSC‐CM inhibits the malignant properties of glioma cells are unclear. This aspect should be explored comprehensively in future studies. Research into the antitumour effects of NSCs is ongoing, and much remains to be elucidated. The antiglioma factors released from different NSCs warrant further validation using multi‐omics technologies.

## Conclusion

We evaluated the effects of human NSCs on glioma cell progression via their paracrine activities. Our findings suggest that NSC‐derived factors can inhibit glioma cell proliferation, invasion, migration and cell cycle progression *in vitro*, while also inducing apoptosis. IFN‐α and DKK1 were secreted into human and rat NSC‐CM and possibly contributed to the antitumour effects by inactivating the Wnt/β‐catenin signalling pathway. We revealed that hNSC‐CM at least partially reduces the proliferation and invasion potential of three glioma cell lines through the Wnt/β‐catenin pathway. Overall, the current findings highlight the great potential of NSC‐derived factors as vehicles for therapeutic agents or targets for the clinical treatment of glioma.

## Conflict of interest

The authors declare no conflicts of interest.

## Author contributions

JL conceived the project. YL and LW supervised this project. XY performed the majority of the experiments. XX, KY, WC and CH provided intellectual input. XY and XL wrote the manuscript.

## Data Availability

All data generated or analysed during this study are included in this published article.

## References

[1] Götz M and Huttner WB (2005) The cell biology of neurogenesis. Nat Rev Mol Cell Biol 6, 777–788. 16314867 10.1038/nrm1739

[2] Wang J , Liu J , Meng H , Guan Y , Yin Y , Zhao Z , Sun G , Wu A , Chen L and Yu X (2019) Neural stem cells promote glioblastoma formation in nude mice. Clin Transl Oncol 21, 1551–1560. 30945128 10.1007/s12094-019-02087-x

[3] Bagó JR , Sheets KT and Hingtgen SD (2016) Neural stem cell therapy for cancer. Methods 99, 37–43. 26314280 10.1016/j.ymeth.2015.08.013 PMC5436904

[4] Walker T , Huang J and Young K (2016) Neural stem and progenitor cells in nervous system function and therapy. Stem Cells Int 2016, 1890568. 27579042 10.1155/2016/1890568 PMC4989086

[5] Borhani‐Haghighi M and Mohamadi Y (2021) The protective effects of neural stem cells and neural stem cells‐conditioned medium against inflammation‐induced prenatal brain injury. J Neuroimmunol 360, 577707. 34507013 10.1016/j.jneuroim.2021.577707

[6] Chen T , Li Y , Ni W , Tang B , Wei Y , Li J , Yu J , Zhang L , Gao J , Zhou J *et al*. (2020) Human neural stem cell‐conditioned medium inhibits inflammation in macrophages via Sirt‐1 signaling pathway in vitro and promotes sciatic nerve injury recovery in rats. Stem Cells Dev 29, 1084–1095. 32560594 10.1089/scd.2020.0020

[7] Baulch JE , Acharya MM , Allen BD , Ru N , Chmielewski NN , Martirosian V , Giedzinski E , Syage A , Park AL , Benke SN *et al*. (2016) Cranial grafting of stem cell‐derived microvesicles improves cognition and reduces neuropathology in the irradiated brain. Proc Natl Acad Sci USA 113, 4836–4841. 27044087 10.1073/pnas.1521668113 PMC4855546

[8] Cheng Z , Bosco DB , Sun L , Chen X , Xu Y , Tai W , Didier R , Li J , Fan J , He X *et al*. (2017) Neural stem cell‐conditioned medium suppresses inflammation and promotes spinal cord injury recovery. Cell Transplant 26, 469–482. 27737726 10.3727/096368916X693473 PMC5657700

[9] Liang P , Liu J , Xiong J , Liu Q , Zhao J , Liang H , Zhao L and Tang H (2014) Neural stem cell‐conditioned medium protects neurons and promotes propriospinal neurons relay neural circuit reconnection after spinal cord injury. Cell Transplant 23 (Suppl 1), S45–S56. 25333841 10.3727/096368914X684989

[10] Louis DN , Perry A , Wesseling P , Brat DJ , Cree IA , Figarella‐Branger D , Hawkins C , Ng HK , Pfister SM , Reifenberger G *et al*. (2021) The 2021 WHO classification of tumors of the central nervous system: a summary. Neuro Oncol 23, 1231–1251. 34185076 10.1093/neuonc/noab106 PMC8328013

[11] Ostrom QT , Cioffi G , Waite K , Kruchko C and Barnholtz‐Sloan JS (2021) CBTRUS statistical report: primary brain and other central nervous system tumors diagnosed in the United States in 2014–2018. Neuro Oncol 23, iii1–iii105. 34608945 10.1093/neuonc/noab200 PMC8491279

[12] Calinescu AA , Kauss MC , Sultan Z , Al‐Holou WN and O'Shea SK (2021) Stem cells for the treatment of glioblastoma: a 20‐year perspective. CNS Oncologia 10, CNS73. 10.2217/cns-2020-0026 PMC8162173 34006134

[13] Yuan X , Hu J , Belladonna ML , Black KL and Yu JS (2006) Interleukin‐23‐expressing bone marrow‐derived neural stem‐like cells exhibit antitumor activity against intracranial glioma. Cancer Res 66, 2630–2638. 16510582 10.1158/0008-5472.CAN-05-1682

[14] Hao SC , Ma H , Niu ZF , Sun SY , Zou YR and Xia HC (2019) hUC‐MSCs secreted exosomes inhibit the glioma cell progression through PTENP1/miR‐10a‐5p/PTEN pathway. Eur Rev Med Pharmacol Sci 23, 10013–10023. 31799671 10.26355/eurrev_201911_19568

[15] Bagó JR , Okolie O , Dumitru R , Ewend MG , Parker JS , Werff RV , Underhill TM , Schmid RS , Miller CR and Hingtgen SD (2017) Tumor‐homing cytotoxic human induced neural stem cells for cancer therapy. Sci Transl Med 9, eaah6510. 28148846 10.1126/scitranslmed.aah6510 PMC6719790

[16] Mooney R , Hammad M , Batalla‐Covello J , Abdul Majid A and Aboody KS (2018) Concise review: neural stem cell‐mediated targeted cancer therapies. Stem Cells Transl Med 7, 740–747. 30133188 10.1002/sctm.18-0003 PMC6186269

[17] Zhang Q , Xiang W , Yi DY , Xue BZ , Wen WW , Abdelmaksoud A , Xiong NX , Jiang XB , Zhao HY and Fu P (2018) Current status and potential challenges of mesenchymal stem cell‐based therapy for malignant gliomas. Stem Cell Res Ther 9, 228. 30143053 10.1186/s13287-018-0977-z PMC6109313

[18] Yang C , Lei D , Ouyang W , Ren J , Li H , Hu J and Huang S (2014) Conditioned media from human adipose tissue‐derived mesenchymal stem cells and umbilical cord‐derived mesenchymal stem cells efficiently induced the apoptosis and differentiation in human glioma cell lines in vitro. Biomed Res Int 2014, 109389. 24971310 10.1155/2014/109389 PMC4058294

[19] Cortes‐Dericks L , Froment L , Kocher G and Schmid RA (2016) Human lung‐derived mesenchymal stem cell‐conditioned medium exerts in vitro antitumor effects in malignant pleural mesothelioma cell lines. Stem Cell Res Ther 7, 25. 26861734 10.1186/s13287-016-0282-7 PMC4748521

[20] Zhao W , Ren G , Zhang L , Zhang Z , Liu J , Kuang P , Yin Z and Wang X (2012) Efficacy of mesenchymal stem cells derived from human adipose tissue in inhibition of hepatocellular carcinoma cells in vitro. Cancer Biother Radiopharm 27, 606–613. 22917212 10.1089/cbr.2011.1150

[21] Parsons MJ , Tammela T and Dow LE (2021) WNT as a driver and dependency in cancer. Cancer Discov 11, 2413–2429. 34518209 10.1158/2159-8290.CD-21-0190 PMC8487948

[22] He S and Tang S (2020) WNT/β‐catenin signaling in the development of liver cancers. Biomed Pharmacother 132, 110851. 33080466 10.1016/j.biopha.2020.110851

[23] Zhang N , Wei P , Gong A , Chiu WT , Lee HT , Colman H , Huang H , Xue J , Liu M , Wang Y *et al*. (2011) FoxM1 promotes β‐catenin nuclear localization and controls Wnt target‐gene expression and glioma tumorigenesis. Cancer Cell 20, 427–442. 22014570 10.1016/j.ccr.2011.08.016 PMC3199318

[24] Gong A and Huang S (2012) FoxM1 and Wnt/β‐catenin signaling in glioma stem cells. Cancer Res 72, 5658–5662. 23139209 10.1158/0008-5472.CAN-12-0953 PMC3500394

[25] Pu P , Zhang Z , Kang C , Jiang R , Jia Z , Wang G and Jiang H (2009) Downregulation of Wnt2 and beta‐catenin by siRNA suppresses malignant glioma cell growth. Cancer Gene Ther 16, 351–361. 18949017 10.1038/cgt.2008.78

[26] Jin X , Jeon HY , Joo KM , Kim JK , Jin J , Kim SH , Kang BG , Beck S , Lee SJ , Kim JK *et al*. (2011) Frizzled 4 regulates stemness and invasiveness of migrating glioma cells established by serial intracranial transplantation. Cancer Res 71, 3066–3075. 21363911 10.1158/0008-5472.CAN-10-1495

[27] Li X , Tan R , Hu X , Jiao Q , Rahman MS , Chen X , Zhang P , An J , Lu H and Liu Y (2019) Neural stem cell‐derived factors inhibit the growth and invasion of U87 stem‐like cells in vitro. J Cell Biochem 120, 5472–5479. 30367517 10.1002/jcb.27826

[28] Wei WJ , Wang YC , Guan X , Chen WG and Liu J (2022) A neurovascular unit‐on‐a‐chip: culture and differentiation of human neural stem cells in a three‐dimensional microfluidic environment. Neural Regen Res 17, 2260–2266. 35259847 10.4103/1673-5374.337050 PMC9083144

[29] Wang Y , Ma J , Li N , Wang L , Shen L , Sun Y , Wang Y , Zhao J , Wei W , Ren Y *et al*. (2017) Microfluidic engineering of neural stem cell niches for fate determination. Biomicrofluidics 11, 014106. 28798841 10.1063/1.4974902 PMC5533482

[30] Xu Y , Zhou J , Li L , Yang W , Zhang Z , Zhang K , Ma K , Xie H , Zhang Z , Cai L *et al*. (2022) FTO‐mediated autophagy promotes progression of clear cell renal cell carcinoma via regulating SIK2 mRNA stability. Int J Biol Sci 18, 5943–5962. 36263177 10.7150/ijbs.77774 PMC9576516

[31] Roh H , Kim H and Park JK (2021) Construction of a fibroblast‐associated tumor spheroid model based on a collagen drop array chip. Biosensors 11, 506. 34940263 10.3390/bios11120506 PMC8699288

[32] Liu X , Chen D , Chen H , Wang W , Liu Y , Wang Y , Duan C , Ning Z , Guo X , Otkur W *et al*. (2021) YB1 regulates miR‐205/200b‐ZEB1 axis by inhibiting microRNA maturation in hepatocellular carcinoma. Cancer Commun (Lond) 41, 576–595. 34110104 10.1002/cac2.12164 PMC8286141

[33] Červenka J , Tylečková J , Kupcová Skalníková H , Vodičková Kepková K , Poliakh I , Valeková I , Pfeiferová L , Kolář M , Vaškovičová M , Pánková T *et al*. (2020) Proteomic characterization of human neural stem cells and their secretome during in vitro differentiation. Front Cell Neurosci 14, 612560. 33584205 10.3389/fncel.2020.612560 PMC7876319

[34] Dause TJ , Denninger JK , Smith BM and Kirby ED (2022) The neural stem cell secretome across neurodevelopment. Exp Neurol 355, 114142. 35709983 10.1016/j.expneurol.2022.114142

[35] Hinshaw DC and Shevde LA (2019) The tumor microenvironment innately modulates cancer progression. Cancer Res 79, 4557–4566. 31350295 10.1158/0008-5472.CAN-18-3962 PMC6744958

[36] Pituch KC , Zannikou M , Ilut L , Xiao T , Chastkofsky M , Sukhanova M , Bertolino N , Procissi D , Amidei C , Horbinski CM *et al*. (2021) Neural stem cells secreting bispecific T cell engager to induce selective antiglioma activity. Proc Natl Acad Sci USA 118, e2015800118. 33627401 10.1073/pnas.2015800118 PMC7936285

[37] Chen FX , Ren WW , Yang Y , Shen D , Zong Y , Xu S , Duan Y , Qian Y and Ji Y (2009) Reciprocal effects of conditioned medium on cultured glioma cells and neural stem cells. J Clin Neurosci 16, 1619–1623. 19836246 10.1016/j.jocn.2009.04.009

[38] Qian C , Wang Y , Ji Y , Chen D , Wang C , Zhang G and Wang Y (2022) Neural stem cell‐derived exosomes transfer miR‐124‐3p into cells to inhibit glioma growth by targeting FLOT2. Int J Oncol 61, 115. 35929514 10.3892/ijo.2022.5405 PMC9387557

[39] Li Z , Zhong Q , Liu H , Liu P , Wu J , Ma D , Chen X and Yang X (2016) Conditioned medium from neural stem cells inhibits glioma cell growth. Cell Mol Biol (Noisy‐le‐Grand) 62, 68–73. 10.14715/cmb/2016.62.12.12 27894403

[40] An J , Yan H , Li X , Tan R , Chen X , Zhang Z , Liu Y , Zhang P , Lu H and Liu Y (2017) The inhibiting effect of neural stem cells on proliferation and invasion of glioma cells. Oncotarget 8, 76949–76960. 29100360 10.18632/oncotarget.20270 PMC5652754

[41] Lee JH , Lee JE , Kahng JY , Kim SH , Park JS , Yoon SJ , Um JY , Kim WK , Lee JK , Park J *et al*. (2018) Human glioblastoma arises from subventricular zone cells with low‐level driver mutations. Nature 560, 243–247. 30069053 10.1038/s41586-018-0389-3

[42] Boon EMJ , van der Neut R , van de Wetering M , Clevers H and Pals ST (2002) Wnt signaling regulates expression of the receptor tyrosine kinase met in colorectal cancer. Cancer Res 62, 5126–5128. 12234972

[43] Birchmeier C , Birchmeier W , Gherardi E and Vande Woude GF (2003) Met, metastasis, motility and more. Nat Rev Mol Cell Biol 4, 915–925. 14685170 10.1038/nrm1261

[44] Fu M , Wang C , Li Z , Sakamaki T and Pestell RG (2004) Minireview: cyclin D1: normal and abnormal functions. Endocrinology 145, 5439–5447. 15331580 10.1210/en.2004-0959

[45] Ranuncolo SM , Ladeda V , Specterman S , Varela M , Lastiri J , Morandi A , Matos E , de Kier B , Joffé E , Puricelli L *et al*. (2002) CD44 expression in human gliomas. J Surg Oncol 79, 30–35; discussion 35–36. 11754374 10.1002/jso.10045

[46] Llombart V and Mansour MR (2022) Therapeutic targeting of “undruggable” MYC. EBioMedicine 75, 103756. 34942444 10.1016/j.ebiom.2021.103756 PMC8713111

[47] Pelengaris S , Khan M and Evan G (2002) c‐MYC: more than just a matter of life and death. Nat Rev Cancer 2, 764–776. 12360279 10.1038/nrc904

[48] Martin FT , Dwyer RM , Kelly J , Khan S , Murphy JM , Curran C , Miller N , Hennessy E , Dockery P , Barry FP *et al*. (2010) Potential role of mesenchymal stem cells (MSCs) in the breast tumour microenvironment: stimulation of epithelial to mesenchymal transition (EMT). Breast Cancer Res Treat 124, 317–326. 20087650 10.1007/s10549-010-0734-1

[49] Binello E and Germano IM (2012) Stem cells as therapeutic vehicles for the treatment of high‐grade gliomas. Neuro Oncol 14, 256–265. 22166262 10.1093/neuonc/nor204 PMC3280798

[50] Semënov MV , Tamai K , Brott BK , Kühl M , Sokol S and He X (2001) Head inducer Dickkopf‐1 is a ligand for Wnt coreceptor LRP6. Curr Biol 11, 951–961. 11448771 10.1016/s0960-9822(01)00290-1

[51] Ma S , Liang S , Jiao H , Chi L , Shi X , Tian Y , Yang B and Guan F (2014) Human umbilical cord mesenchymal stem cells inhibit C6 glioma growth via secretion of dickkopf‐1 (DKK1). Mol Cell Biochem 385, 277–286. 24104453 10.1007/s11010-013-1836-y

[52] Jia Y , Chen L , Guo S and Li Y (2019) Baicalin induced colon cancer cells apoptosis through miR‐217/DKK1‐mediated inhibition of Wnt signaling pathway. Mol Biol Rep 46, 1693–1700. 30737617 10.1007/s11033-019-04618-9

[53] Yu R , Zhu B and Chen D (2022) Type I interferon‐mediated tumor immunity and its role in immunotherapy. Cell Mol Life Sci 79, 191. 35292881 10.1007/s00018-022-04219-z PMC8924142

[54] Zhou L , Zhang Y , Wang Y , Zhang M , Sun W , Dai T , Wang A , Wu X , Zhang S , Wang S *et al*. (2020) A dual role of type I interferons in antitumor immunity. Adv Biosyst 4, e1900237. 33245214 10.1002/adbi.201900237

[55] Alcantara Llaguno S , Chen J , Kwon CH , Jackson EL , Li Y , Burns DK , Alvarez‐Buylla A and Parada LF (2009) Malignant astrocytomas originate from neural stem/progenitor cells in a somatic tumor suppressor mouse model. Cancer Cell 15, 45–56. 19111880 10.1016/j.ccr.2008.12.006 PMC2650425

[56] Liu C , Sage JC , Miller MR , Verhaak RGW , Hippenmeyer S , Vogel H , Foreman O , Bronson RT , Nishiyama A , Luo L *et al*. (2011) Mosaic analysis with double markers reveals tumor cell of origin in glioma. Cell 146, 209–221. 21737130 10.1016/j.cell.2011.06.014 PMC3143261

[57] Takei Y (2019) Age‐dependent decline in neurogenesis of the hippocampus and extracellular nucleotides. Hum Cell 32, 88–94. 30730038 10.1007/s13577-019-00241-9

[58] Apple DM , Solano‐Fonseca R and Kokovay E (2017) Neurogenesis in the aging brain. Biochem Pharmacol 141, 77–85. 28625813 10.1016/j.bcp.2017.06.116

